# Supplementary material for: capTEs enables locus-specific dissection of transcriptional outputs from reference and nonreference transposable elements
Source: Commun Biol. 2023 Sep 23;6:974. doi: 10.1038/s42003-023-05349-1 (PMC10517987; doi:10.1038/s42003-023-05349-1)
Supplement: Supplementary file 2 — Supplementary Information [file 42003_2023_5349_MOESM2_ESM.pdf]

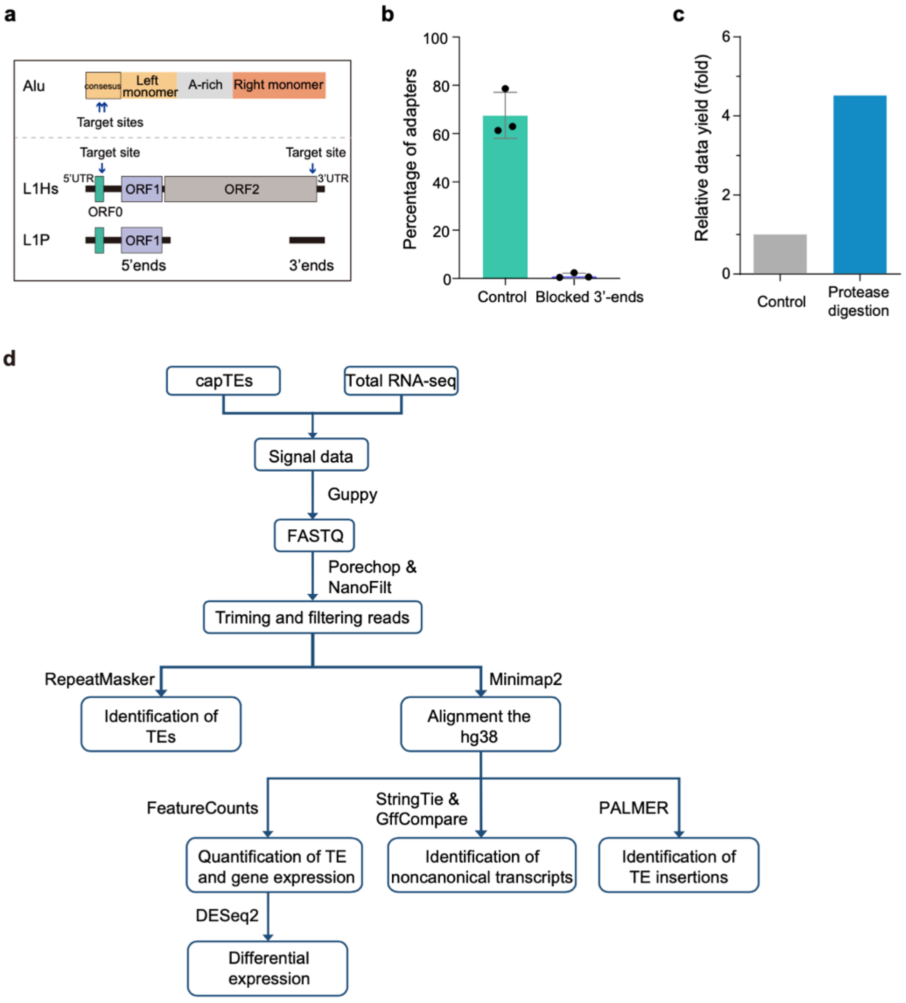

Supplementary Figure 1 Development of capTEs. a, gRNA schematic. Two gRNAs (up arrows) target the consensus region of Alu elements, and two gRNAs (down arrows) target the 5'-ends and 3'-ends of L1 elements. **b,** Bar plot showing the percentage of pores occupied by adapters among all sequencing pores. Blocked 3'-ends (purple, n=3) represent DNA 3'-ends that are blocked by ddGMP tailing. In the control (green, n=3), nCATS is directly applied to capture TE transcripts. Error bars represent standard deviation. **c,** Bar plot showing the improvement in data yield by protease treatment. The data yield in the control (gray, nCATS is directly applied to capture TE transcripts) is normalized to 1. **d.** Workflow for data analysis. The mapping results were obtained from raw signal data using the following steps: base-calling and FASTQ file generation with Guppy, trimming and filtering with Porechop and NanoFilt, alignment to the reference genome and transcriptome (hg38) using minimap2, and TE annotation using RepeatMasker. These results were then utilized for subsequent analysis, including quantifying the expression of TEs and genes, identifying noncanonical transcripts and detecting nonreference TE insertions.

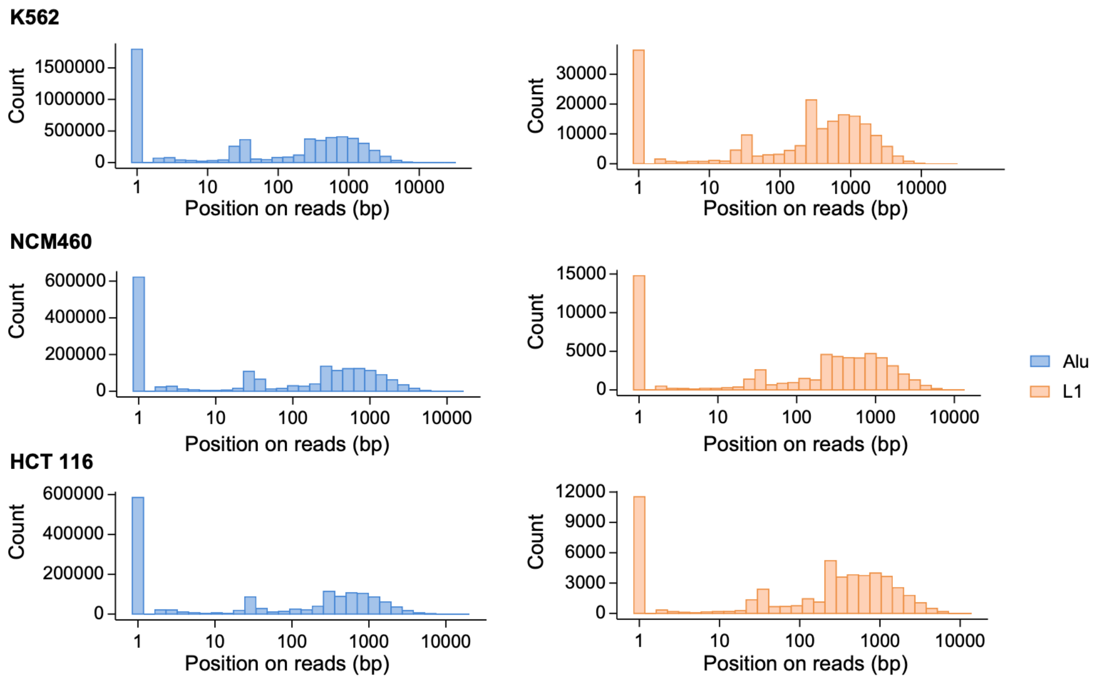

Supplementary Figure 2 Positional distributions of Alu and L1 on reads in K562, NCM460, and HCT 116 cells. Alu and L1 represent TEs targeted by designed gRNAs.

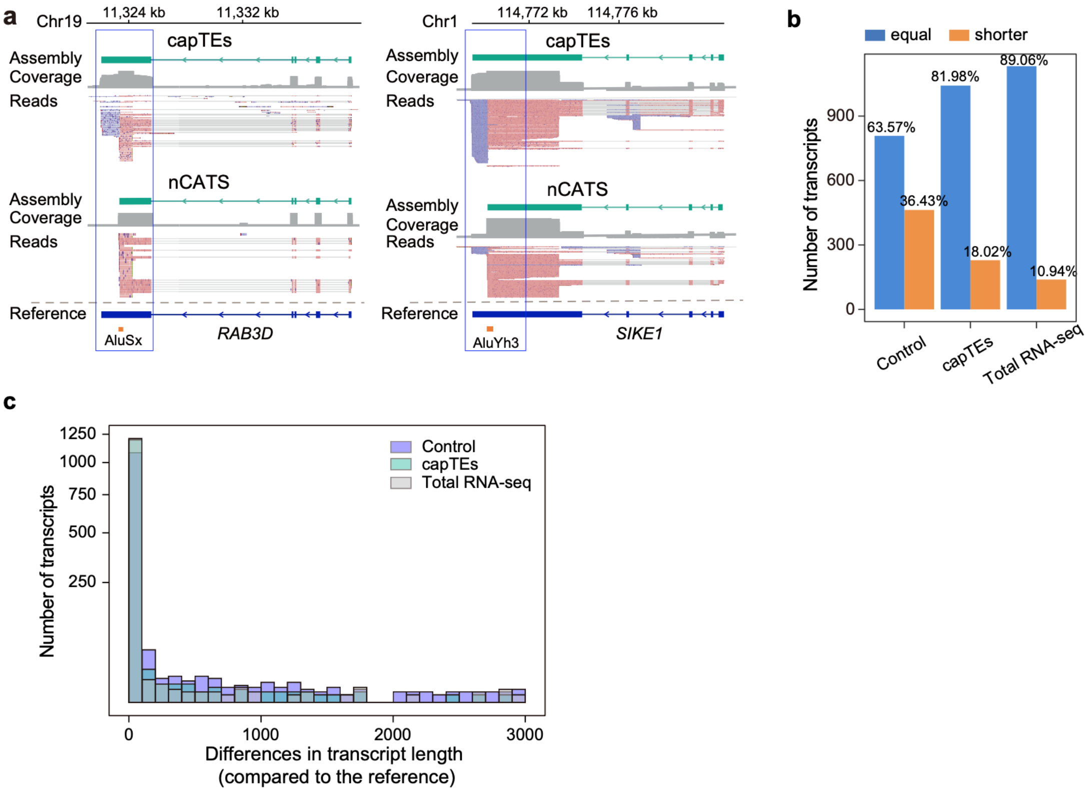

Supplementary Figure 3 The transcript completeness of control, capTEs, and total RNA-seq assemblies. a, Genome browser view showing two examples of transcript assembly of capTEs and nCATS data. The blue rectangle marks the incomplete assembly of nCATS data. **b,** Bar plot showing the number of full-length (blue) and incomplete (orange) transcripts identified by control, capTEs and total RNA-seq methods. Transcripts identified by these three methods and annotated in Gencode were included in this analysis. **c,** Differences in transcript lengths between control (purple), capTEs (blue) and total RNA-seq (gray) and the reference. In the control, nCATS is directly applied to capture TE transcripts.

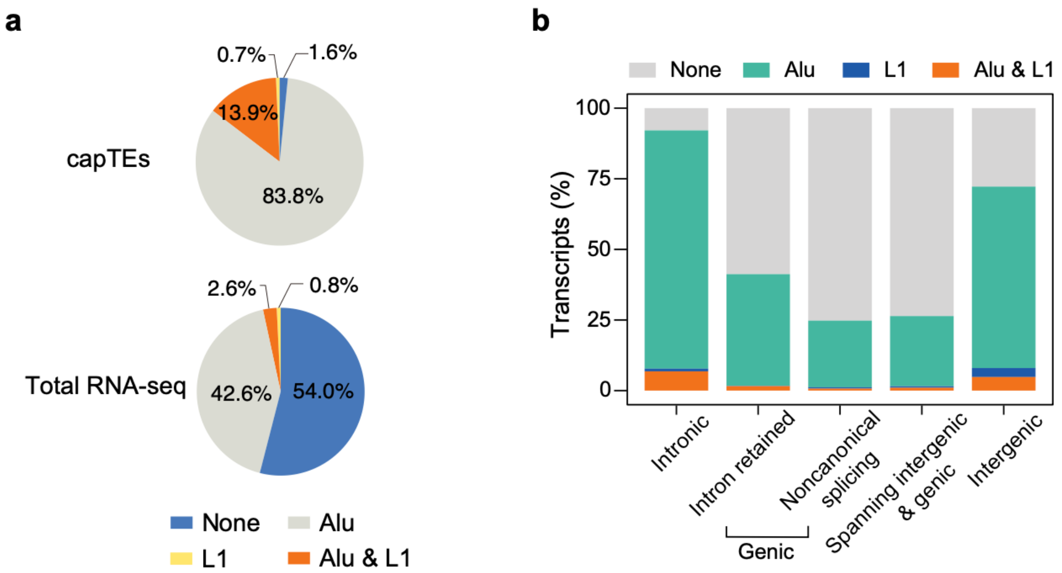

Supplementary Figure 4 The proportion of noncanonical transcripts overlapping with target TEs.

a, Pie charts showing the proportion of noncanonical transcripts that exclusively overlap with Alu (gray), exclusively overlap with L1 (yellow) and overlap with both Alu and L1 (orange) as identified by capTEs and total RNA-seq, respectively. **b,** Stacked bar plot showing the percentage of noncanonical transcripts that exclusively overlap with Alu (green), exclusively overlap with L1 (blue) and overlap both Alu and L1 (orange) among each type of noncanonical transcripts detected by total RNA-seq.

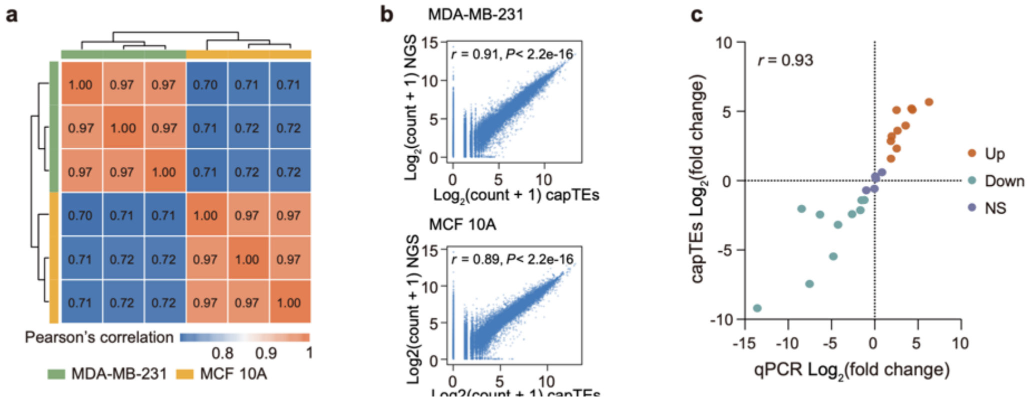

Supplementary Figure 5 The reliability assessment of gene expression levels measured by capTEs. a, Heatmap showing the Pearson's correlation of the expression levels of TE-containing transcripts among three biological replicates of MCF 10A and MDA-MB-231 cells. **b,** Scatter plots showing the Pearson's correlation of expression levels of TE-containing transcripts measured using capTEs and NGS methods. **c,** Scatter plot showing the Pearson's correlation of fold changes in the expression of TE-hosting genes measured by capTEs and qPCR methods. The orange, gray–green and gray–purple dots indicate overexpressed, underexpressed and not significantly changed genes in capTEs results, respectively. *r*: Pearson's correlation coefficient. **a-b,** TE refers to the Alu and L1 elements targeted by our gRNA pool.

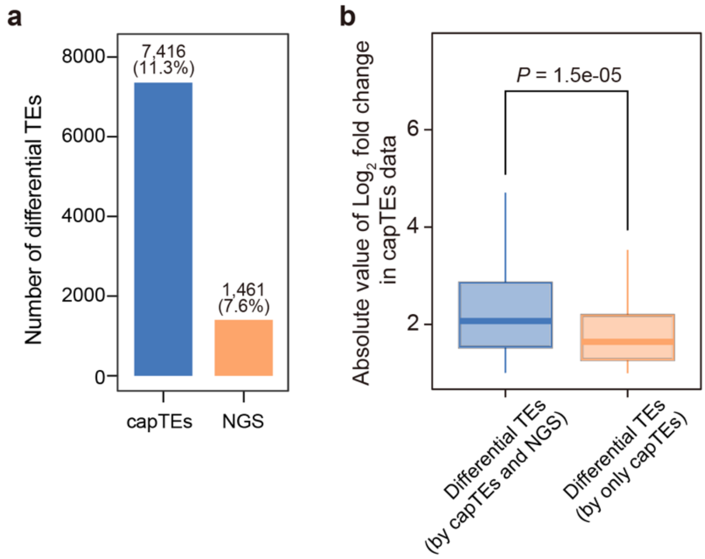

Supplementary Figure 6 Differentially expressed TEs detected with capTEs and NGS methods. a, Bar plot showing the number of differential TE loci measured by capTEs and NGS. The percentages within the parentheses represent the proportion of differential TEs among all the target TEs quantified by the respective method. **b,** Box plot showing the expression fold changes of differential TEs codetected by capTEs and NGS (blue) and differential TEs detected only by capTEs (orange). The *y*-axis represents absolute values of log2 expression fold change determined using capTEs data. The box edges and whiskers indicate the median, upper and lower quartiles (the 25th and 75th percentiles) and 1.5 × interquartile range, respectively. The statistical analysis was performed using Wilcox rank sum test. **a-b,** NGS data were analyzed using Telescope.

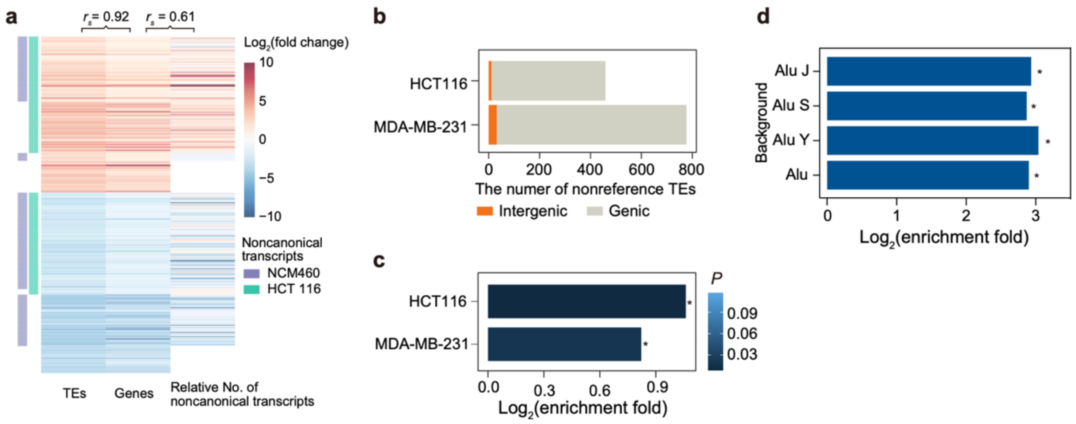

Supplementary Figure 7 TE expression changes and identified transcribed TE insertions in cancer cells. a, Heatmap depicting the correlation among expression changes of target TEs, expression changes of TE-hosting genes and relative number of noncanonical transcripts in HCT 116 cells compared with NCM460 cells. r_s represents Spearman's correlation coefficient. **b,** Stacked bar plot showing the number of Alu and L1 insertions in genic (gray) and intergenic (orange) regions in cancer cells HCT 116 and MDA-MB-231. **c,** Enrichment analysis of insertional events on oncogenes in breast and colorectal cancer cells. Significant * represents BH-adjusted *P* value < 0.05 reported by the Chi-squared test between inserted TEs and all expressed target TEs. **d,** Location enrichment of Alu insertions in 3'UTRs compared to reference annotated Alu as a whole or Alu major subfamilies, including Alu Y, Alu S and Alu J. All Alu insertions identified in breast and colorectal cancer cells are included and the reference Alu refers to those successfully transcribed. Significant * represents BH-adjusted *P* < 0.05 reported by the Chi-squared test between Alu insertions and all expressed loci of Alu elements or specific subfamilies.

Supplementary Tables

Supplementary Table 1. Cell lines, oligos, gRNAs and synthesized TE cDNAs used in this study.

**Cell lines**		
Cell lines	Sources	
K562	ATCC	
HCT 116	ATCC	
NCM460	INCELL	
MDA-MB-231	ATCC	
MCF-10A	ATCC	
**Oligos for constructing full-length cDNA library**		
Oligos	Sequence (5'→3')	
oligo-dT	ACGAGCATCAGCAGCATACGANNNNNNNNTTTTTTTTTTTTTTTTTTTTTTTTTTTTTVN	
template-switching oligo	AGAGACAGATTGCGCAATGNNNNNNNN/i2OMeG//i2OMeG//i2OMeG/	
cDNA amplification-F	AGAGACAGATTGCGCAATG	
cDNA amplification-R	ACGAGCATCAGCAGCATACGA	
**Oligos for qPCR**		
Gene	F (5'→3')	R (5'→3')
FAM83B	CAGCGTCTCAGGAATATTCGAGTG	GCTGAGGTGAGCTTTCTCAAATGAC
SULT1E1	GCAAGGACAGGTTCCTTATGGTTC	AGCTCCTCTGATGGCTTCCTTTC
ZNF483	CACTGTTTGTCCCAATACTGAGTCC	AAGTCCAGAAATTCTAGGTTCCTGAGG
EPGN	ACAGAAGCTGACAACATAGAAGGACC	AACACCTGCAGATGGCTTTCTC
FKBP5	GAGCTTCGAAAAGGCCAAAGAATCC	TGCCTCCCTTGAAGTATACGGTTC
NF2	AGAAGGCTAGAAAGCAGATGGAGC	ATCAGTGCTTCGTTGGCCATTG
DIXDC1	GATAAAGGATGCCTTGCAGCAGAG	GATCCACATTCTGGTTGTGCAGC
PRKX	AGATGCTTTCGGGGTTTCCTCC	ATCATTCGCCCCGTTCTTCATG
ZNF69	GGAAGTGATGCTGGAAACTTTCAGG	GAGACTCCTGAAGTTTCTCCTGGG
ZNF780B	ACAGCCACCTGATATCACTGGGAA	GACTCCAAATCTGGATACCATCTGCT
OGFRL1	CATTCAATGGCTTTTCCCCCTGAG	GCCCGAGCAACATTTCCAGT
ITGB3	ACCCCTGCTATGATATGAAGACCAC	CATCACAGACTGTAGCCTGCATG
TRIM34	ATGAAATGGAGTGAGATCTGGAGGC	ACCGGACAGCTGTCAGTTCTCTA
CRISPLD2	ATGTGCACGCACTACACACAGATA	CCAATCCAGTTCCCCTTTGGAG
FBXO27	TCGTGACTTCATTCAGCTGGTGT	CCCACCAGTCAGAGACACAAATCTC
ZNF626	AACCTGGTCTTCCTTGGTATTACTGT	AGAACACATTACTGAGGGTTTGGCT
TNFSF10	ACTCCGTCAGCTCGTTAGAAAGATG	GGAGTTTGGAGAAGACAATGTGTTGC
CCDC69	GGGGACACAGCTATAACTGTCCAG	CCACCTGTTGTGCCCATTTCTTC

CGAS	CAAAAAGTAGCTGGCCTGCTAGCA	CCGCCATGTTTCTTCTTGGAAACC
STING1	AGGGAATTTCAACGTGGCCCA	GGAGAATATACAGCCGCTGGCT
IFNAR1	TTCAAGTTCAGTGGCTCCACGC	CCATCAGATGCTTGTACGCGGA
IFNAR2	TGGCCAGGAATCAGAATCAGCAGAA	CTTAGCGAGACCTTGCCTCAAGAC
IFNGR2	AGGAATCCAACAGGTCAAAGGCC	GGAGGCATCTGCCATTGTTTCG
STAT1	CGGCACCTGCAATTGAAAGAAC	AGAGGTCGTCTCGAGGTCAATTAC
STAT2	GTTACCTGACTCTGGTGGAGCAAC	CGTTTTCAGCTCCTGCTTCAGAC
β-actin	CACCAACTGGGACGACAT	ACAGCCTGGATAGCAACG
FOXRED2 (canonical)	GTGCAATGGCGCAATCTCG	AACTAGCCAGGCGTGGTAG
FOXRED2 (all)	GAAGCAGGACTTGCATAGAAGCT	AGACTTCTTAACAGACTGGACTGC
FOXRED2 (noncanonical)	GACAGGTGGATCACTGAAGAGTTAGT	AGGCGTCTTAGTCTCATTTGT
**Oligos for PCR validation of noncanonical TE transcripts**		
Transcript	F (5'→3')	R (5'→3')
STRG. 9149.1	TGCTTCTTAAGGCCAAGGTG	TGGACTTAGCGATGCACTTC
STRG. 9336.2	AGACGCATATGGGAGTGACTG	AGCTCCTTTTCCACTCGTCTG
STRG. 11616.6	GGAAAGATGGAGCAAGAAGCCG	GTAAATCTAGCACTTTAGGAGGCCG
STRG. 7160.1	GGAGTTCCCCAACTTCAACAGC	GCACTACACCACTTACAATTGCTCC
**gRNAs**		
gRNA	Sequence (5'→3')	
Alu gRNA-1	UCCCAAAGUGCUGGGAUUAC	
Alu gRNA-2	GCCUCGGCCUCCCAAAGUGC	
L1 gRNA-1	UCUGAGAUCAAACUGCAAGG	
L1 gRNA-2	UUUCAUCCAUGUCCCUACAA	
**Synthesized TE cDNAs**		
Spike-in	Dilution fold	Concentration (ng/μL) in stock solution
Spike-in 1	4096	0.002441406
Spike-in 2	512	0.01953125
Spike-in 3	256	0.0390625
Spike-in 4	128	0.078125
Spike-in 5	64	0.15625
Spike-in 6	16	0.625
Spike-in 7	4	2.5
Spike-in 8	1	10

Supplementary Table 2. Subfamily proportions within Alu and L1 elements detected by capTEs and total RNA-seq methods. The subfamily proportion refers to the percentage of a specific subfamily among all the Alu and L1 loci detected by

the corresponding method. The enrichment fold was defined as the ratio of the subfamily proportion detected by capTEs to that detected by total RNA-seq.

**Alu subfamilies**					
Subfamily	capTEs detected loci	Total RNA-seq detected loci	Subfamily proportion (capTEs)	Subfamily proportion (total RNA-seq)	Enrichment fold
AluS	3221541	572830	61.50%	61.45%	1.0
AluY	812884	142934	15.52%	15.33%	1.2
AluJ	933656	164174	17.82%	17.61%	1.0
FAM	2816	430	0.05%	0.05%	1.0
FRAM	52299	8956	1.00%	0.96%	1.0
FLAM	215230	42910	4.11%	4.60%	0.9
**L1 subfamilies**					
Subfamily	capTEs detected loci	Total RNA-seq detected loci	Subfamily proportion (capTEs)	Subfamily proportion (total RNA-seq)	Enrichment fold
L1P	178616	26508	93.93%	92.17%	1.0
L1Hs	11538	2252	6.07%	7.83%	0.8

Supplementary Table 3. Alu and L1 subfamilies quantified by capTEs and NGS methods. NGS data were analyzed using Telescope and TEtranscripts. The subfamily proportion refers to the proportion of a specific subfamily among all the detected TE loci. The enrichment fold was defined as the ratio of the subfamily proportion detected by capTEs to that detected by NGS method.

**Alu subfamilies**								
Subfamily	capTEs		NGS (Telescope)			NGS (TEtranscripts)		
	Detected site	Subfamily proportion	Detected site	Subfamily proportion	Enrichment fold	Detected site	Subfamily proportion	Enrichment fold
AluJ	10323	15.99%	4153	23.53%	0.68	5736	24.89%	0.64
AluS	42372	65.65%	11354	64.32%	1.02	14704	63.80%	1.03
AluY	10453	16.19%	1639	9.29%	1.74	2049	8.89%	1.82
FAM	20	0.03%	15	0.08%	0.36	24	0.10%	0.30
FLAM	1239	1.92%	410	2.32%	0.83	436	1.89%	1.01
FRAM	140	0.22%	81	0.46%	0.47	99	0.43%	0.50
**L1 subfamilies**								
Subfamily	capTEs		NGS (Telescope)			NGS (TEtranscripts)		
	Detected site	Subfamily proportion	Detected site	Subfamily proportion	Enrichment fold	Detected site	Subfamily proportion	Enrichment fold
L1Hs	40	4.28%	4	0.28%	15.47	5	0.22%	19.74
L1P	894	95.72%	1441	99.72%	0.96	2300	99.78%	0.96
