# Supplementary material for: capTEs enables locus-specific dissection of transcriptional outputs from reference and nonreference transposable elements
Source: Commun Biol. 2023 Sep 23;6:974. doi: 10.1038/s42003-023-05349-1 (PMC10517987; doi:10.1038/s42003-023-05349-1)
Supplement: Supplementary file 3 — Description of Additional Supplementary Files [file 42003_2023_5349_MOESM3_ESM.pdf]

Description of Additional Supplementary Files

File name: Supplementary Data 1
Description: All branched subfamilies of Alu and L1 elements detected by capTEs and total RNA-seq methods.

File name: Supplementary Data 2
Description: The noncanonical transcripts of K562 cells identified by capTEs and total RNA-seq methods

File name: Supplementary Data 3
Description: The TE insertions identified in K562 cells by capTEs, total RNA-seq and 30× nanopore WGS methods.

File name: Supplementary Data 4
Description: The expression changes of genes and TEs in MDA-MB-231 cells compared to the matched control cells MCF 10A identified using capTEs or NGS-based methods.

File name: Supplementary Data 5
Description: The noncanonical transcripts identified using capTEs in MDA-MB-231, MCF 10A, HCT 116 and NCM460 cells.

File name: Supplementary Data 6
Description: The expression changes of genes and TEs identified using capTEs in HCT 116 cells compared to the matched control cells NCM460.

File name: Supplementary Data 7
Description: The TE insertions identified by capTEs in MDA-MB-231, MCF 10A, HCT 116 and NCM460 cells.

File name: Supplementary Data 8
Description: The source data for Figures 1-5.
